# Multi-Omics Integrative Analysis of Lung Adenocarcinoma: An *in silico* Profiling for Precise Medicine

**DOI:** 10.3389/fmed.2022.894338

**Published:** 2022-06-03

**Authors:** Xinjia Ruan, Yuqing Ye, Wenxuan Cheng, Li Xu, Mengjia Huang, Yi Chen, Junkai Zhu, Xiaofan Lu, Fangrong Yan

**Affiliations:** State Key Laboratory of Natural Medicines, Research Center of Biostatistics and Computational Pharmacy, China Pharmaceutical University, Nanjing, China

**Keywords:** lung adenocarcinoma, molecular classification, multi-omics data, immunotherapy, precision medicine

## Abstract

Lung adenocarcinoma (LUAD) is one of the most common histological subtypes of lung cancer. The aim of this study was to construct consensus clusters based on multi-omics data and multiple algorithms. In order to identify specific molecular characteristics and facilitate the use of precision medicine on patients we used gene expression, DNA methylation, gene mutations, copy number variation data, and clinical data of LUAD patients for clustering. Consensus clusters were obtained using a consensus ensemble of five multi-omics integrative algorithms. Four molecular subtypes were identified. The CS1 and CS2 subtypes had better prognosis. Based on the immune and drug sensitivity predictions, we inferred that CS1 may be less responsive to immunotherapy and less sensitive to chemotherapeutic drugs. The high immune infiltration of CS2 cells may respond well to immunotherapy. Additionally, the CS2 subtype may also respond to EGFR molecular targeted therapy. The CS3 and CS4 subtypes were associated with poor prognosis. These two subtypes had more mutations, especially TP53 ones, as well as higher sensitivity to chemotherapeutics for lung cancer. However, CS3 was enriched in immune-related pathways and may respond to anti-PD1 immunotherapy. In addition, CS1 and CS4 were less sensitive to ferroptosis inhibitors. We performed a comprehensive analysis of the five types of omics data using five clustering algorithms to reveal the molecular characteristics of LUAD patients. These findings provide new insights into LUAD subtypes and potential clinical treatment strategies to guide personalized management and treatment.

## Introduction

Non-small cell lung cancer (NSCLC) accounts for more than 80% of lung cancer cases and is the second most common cancer worldwide, with a 5-year survival rate of ~16% (1). Within the aforementioned percentage, 45% accounts for lung squamous cell carcinoma (LUSC) cases and 30% accounts for lung adenocarcinoma (LUAD) cases (2). Many epidemiological and experimental studies have attributed the occurrence and progression of LUAD mainly to environmental factors and genetic alterations (3, 4). Recent multi-omics studies have shown that there are significant differences in the copy number variation (CNV) and methylation of LUAD subtypes with different prognoses (5, 6), and the low expression of CNTN4 and RFTN1 predicts poorer clinical outcomes in LUAD patients (7). Mutations such as EGFR, KRAS, and TP53 play an important role in lung tumorigenesis (8); however, not all tumors develop only by activating these mutations and are eliminated by suppressing these genes. The occurrence and development of lung cancer is a complex dynamic process that relies on synergistic interactions among genes, mutations, and the tumor microenvironment. Multi-omics analysis can reveal synergistic interactions, and a subset of genes identified from different histological studies is closely related to biological functions (9). Therefore, it is important to analyze epigenetics, mutations, and transcriptomes using comprehensive multi-omics analyses. Histological and genetic diversity can explain some individual differences in LUAD. Moreover, the identification of the molecular subtypes of LUAD will facilitate the implementation of precision medicine and improve patient prognosis.

**Figure 1:**
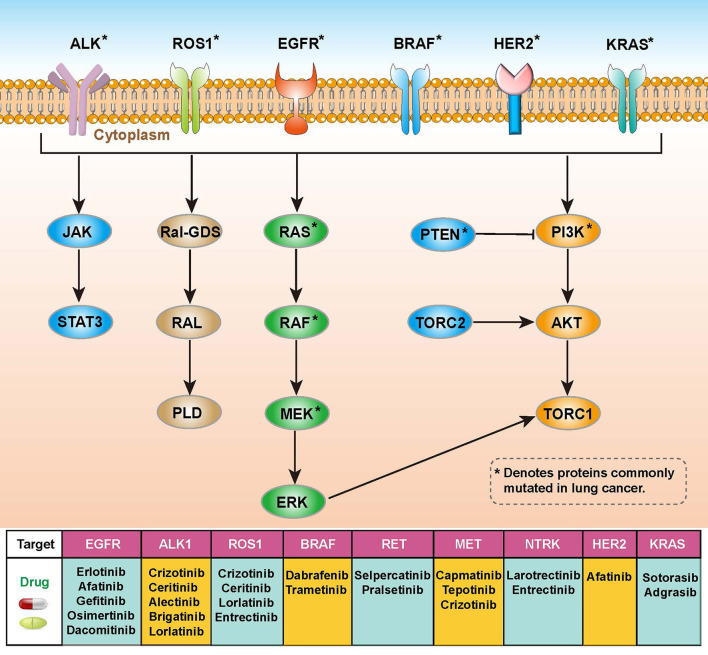
Overview of molecular pathways, potential targets and drugs in non-small cell lung cancer (NSCLC).

Lung cancer is the leading cause of cancer-related mortality and is highly resistant to conventional radiotherapy and chemotherapy (10). In addition to radiotherapy, there are two main types of genetic-related therapeutic strategies, targeted therapy and immunotherapy. Targeted therapies require specific genetic mutations that were harbored by patients with lung cancer. These mutations in receptors or protein kinases can affect related signaling pathways such as the RAS-RAF-MEK-ERK, PI3K-AKT-mTOR or JAK-STAT pathways, and corresponding drugs using in the targeted therapy have been developed for these targets (Figure 1) (11, 12). Immunotherapy is an effective and safe treatment approach, which is always achieved through the introduction of immune checkpoint blockers (ICBs) such as antibodies targeting programmed death 1 (PD-1) and cytotoxic T-lymphocyte antigen-4 (CTLA-4) (13, 14). However, this approach is heavily influenced by the tumor microenvironment (TME), including T cell abundance and tumor mutational load (15). It is important to gain insight into how TME coordinates treatment and prognostic outcomes. The high heterogeneity and complex molecular patterns of LUAD limit the benefit of targeted therapies for specific patients. Therefore, a more comprehensive understanding of the molecular mechanisms of LUAD is necessary to develop precise treatments and to identify the populations that will benefit most from it.

The underlying molecular mechanisms of tumors can help identify a range of prognostic or diagnostically relevant biomarkers (16). Although previous studies have made great progress in understanding the pathogenesis and treatment strategies of LUAD (17, 18), there are shortcomings, such as insufficient omics data or a lack of drug treatment analysis. In this study, we performed a multi-omics analysis of the genomics, epigenomics, and transcriptomics of LUAD. The data were comprehensively analyzed using five clustering algorithms to reveal the molecular features of LUAD patients. Additionally, we discussed potential clinical treatment strategies based on specific molecular features, including drug chemotherapy, immunotherapy, and targeted therapy, which will hopefully be beneficial in guiding the personalized management and treatment of patients.

## Materials and Methods

### Patients and Samples

The sample data were obtained from LUAD patients from The Cancer Genome Atlas (TCGA). We used the “TCGAbiolinks” (19) R package to obtain the transcriptomic expression data of the TCGA-LUAD cohort. Then, we preprocessed the TCGA-based RNA-seq data to filter out low-expressed genes, and only retained genes with a count per million (CPM) ≤ 1 in at least 10% of the samples. Filtered mRNAs were annotated using the GENCODE 27 file. Protein-coding genes were filtered and lncRNAs were identified using Vega (https://vega.archive.ensembl.org/). We then calculated the number of non-overlapping exons per thousand bases per million mapped segments (FPKM) and converted FPKM into transcripts with values per thousand million (TPM). The TPM expression data for mRNA, lncRNA, and miRNA were first transformed by log2 calculations. Methylation data were evaluated by TCGA using the Infinium 450K array, and corresponding clinical data were downloaded from Xena Public Data Hubs (https://xena.ucsc.edu/public-hubs), while somatic mutation data were obtained from Firehose (http://www.firehose.org/). After matching the gene expression, methylation, mutation, copy number variation data, and clinical data of 522 LUAD patients, the multi-omics data of 437 patients were finally included in the follow-up analysis. The basic clinical information of each patient is presented in Supplementary Table S1.

The mRNA expression matrix and clinical information were obtained as validation datasets from external GEO cohorts including GSE68465 (20), GSE72094 (21), and GSE41271 (22). Information on the sample size, platform, and tissue sources of these cohorts is provided in Supplementary Table S2.

### Identification of Molecular Subtypes

We identified subtypes of LUAD patients based on mRNA expression, lncRNA expression, miRNA expression, DNA methylation, and somatic mutation data using the R package “MOVICS” (23). We filtered features that were considered elites, including 1,500 mRNAs, 1,000 lncRNAs, 1,500 miRNAs, 1,500 DNA CpG methylation sites, and mutated genes with mutation rates above 0.3. To determine the appropriate number of subtypes, we analyzed the clustering prediction index (CPI) (24) and Gap-statistics (25) based on multi-omics data. CPI is computed as the average of the adjusted rand indices, while Gap-statistics compares the change in within-cluster dispersion with that expected under an appropriate reference null distribution. The larger the CPI value and the Gap-statistics value, the better the clustering effect. Subsequently, clustering was performed using five advanced multi-omics clustering algorithms: iClusterBayes, SNF, ConsensusClustering, CIMLR, and MoCluster. We listed the specific parameters of each method in Supplementary Table S3. Finally, the combined classification was obtained through the consensus set obtained from the “getConsensusMOIC()” function, and the subtypes were identified with high robustness. Sample similarity in subtypes was quantified using silhouette scores.

### Pathway Enrichment Analysis

Using the raw count data of RNA-seq gene expression, differentially expressed mRNAs were identified by the R package “DESeq2” (26). The filtering parameters for differentially expressed mRNAs were set as follows: false discovery rate (FDR) < 0.05, |log 2 Fold change (log2 FC)| > 2.

We used the R package “clusterProfiler” (27) for gene set enrichment analysis (GSEA), and *P*-values were adjusted for multiple testing using the Benjamini-Hochberg method with a threshold of FDR <0.05. To further reveal the specific features of each subtype, we assessed the activation levels of pathways of interest, including previously published oncogenic pathways (28), metabolic pathways (29), and immune cell features (30). We calculated single-sample gene set enrichment scores for the pathways of interest by the R package “GSVA” (31), and the average enrichment scores for each subtype were used for visualization by a heatmap.

### Characterization of Genetic Alteration on Subtypes

Somatic copy number alteration (SCNA) data were downloaded from Firehose, and SCNA analysis was performed using GISTIC2.0 (32) on GenePattern. We selected the hg19.mat reference genome file for annotation, explored genomic regions with significant amplifications or deletions (threshold of 0.1), and further visualized chromosomal amplifications and deletions at the arm level for analysis.

We calculated differences in mutation frequencies between individual samples using the “maftools” package (33), identified mutations with significant differences (*p* < 0.05), and produced an overall mutation landscape map using Oncoprint.

TMB was assessed by counting the number of non-synonymous mutations per million bases. FGA is the percentage of the genome affected by an increase or decrease in copy numbers.

### Immune Microenvironment Analysis

We evaluated the infiltration rate of immune cells using the CIBERSORT (34) algorithm to estimate the abundance of 22 immune cell types (LM22) between different tumor stages. Additionally, a microenvironmental cell population counter (MCPcounter) (30) was used to estimate the number of infiltrating immune cells. The absolute abundance of eight immune cell types and two stromal cell populations in heterogeneous cellular tissues was quantified based on transcriptomic data, thus reflecting the characteristics of the tumor microenvironment.

### Intra-Tumoral Heterogeneity

We used the mutant-allele tumor heterogeneity (MATH) score by the R package “maftools” to assess intra-tumoral heterogeneity. Each tumor's MATH (35) score was calculated from the median absolute deviation (MAD) and the median of the mutant-allele fractions at the tumor-specific mutated loci. This score quantifies genetic heterogeneity within a tumor using the normalized variance of the frequency distribution of mutant alleles involved in somatic mutations.

### Assessing the Response to Immunotherapy

To assess the likelihood of individual response to immunotherapy, we used the tumor immune dysfunction and exclusion (TIDE) algorithm (36). Higher TIDE scores indicate greater dysfunction and rejection of T cells by the immune microenvironment, suggesting a lower likelihood of benefiting from immune checkpoint blockade (ICB). We used the TIDE web application (http://tide.dfci.harvard.edu/) to analyze the response status of each sample to immunotherapy.

In addition, specific gene sets containing 795 genes were obtained from melanoma cohorts in which patients received anti-CTLA-4 or anti-PD-1 checkpoint inhibition therapy. Unsupervised subclass mapping analysis (SubMap) (37) was used to compare similarities between lung adenocarcinoma and immunotherapy subgroups and to identify responders to anti-CTLA-4 or anti-PD-1 immunotherapy.

### Chemotherapeutic Response Prediction

We predicted the chemotherapeutic response of each sample by the R package “pRRophetic” (38) based on the Genomics of Drug Sensitivity in Cancer (GDSC) database (39). “AllSoldTumours” was selected as the tissue type for analysis, and the batch effect of cell lines was eliminated using the ComBat function. The mean value was used for repeated gene expression measurements, and all other parameters were set to their default values. The maximum half-inhibitory concentration (IC_50_) of the samples was estimated by ridge regression fitting to a homogenized dataset, with a lower IC_50_ indicating higher drug sensitivity. The accuracy of the predictions was assessed using ten-fold cross-validation based on the GDSC training set, resulting in a sensitivity estimate for each chemotherapeutic drug.

### Validation of External Cohorts

We used nearest template prediction (NTP), which can be flexibly applied to cross-platform, cross-species, and multiclass predictions without any optimization of the analysis parameters, to predict subtypes in validation cohorts (40).

### Statistical Analyses

All statistical analyses were performed using R software (version 4.1.1). Fisher's exact test for independence was used to statistically test the association between categorical clinical information and the defined subtypes. Either the chi-squared test or Fisher's exact test was used to test the significance of the logistic regression of categorical data when appropriate. The Wilcoxon test (Mann-Whitney test) was used for continuous data. Survival analysis was performed using the R package survival, and Kaplan-Meier plots and log-rank tests were used to assess the difference in the overall survival (OS) between subtypes (41). For all statistical analyses, *P* < 0.05 were considered statistically significant.

## Results

### The Multi-Omics Classification of LUAD Resulted in Four Subtypes

**Figure 2:**
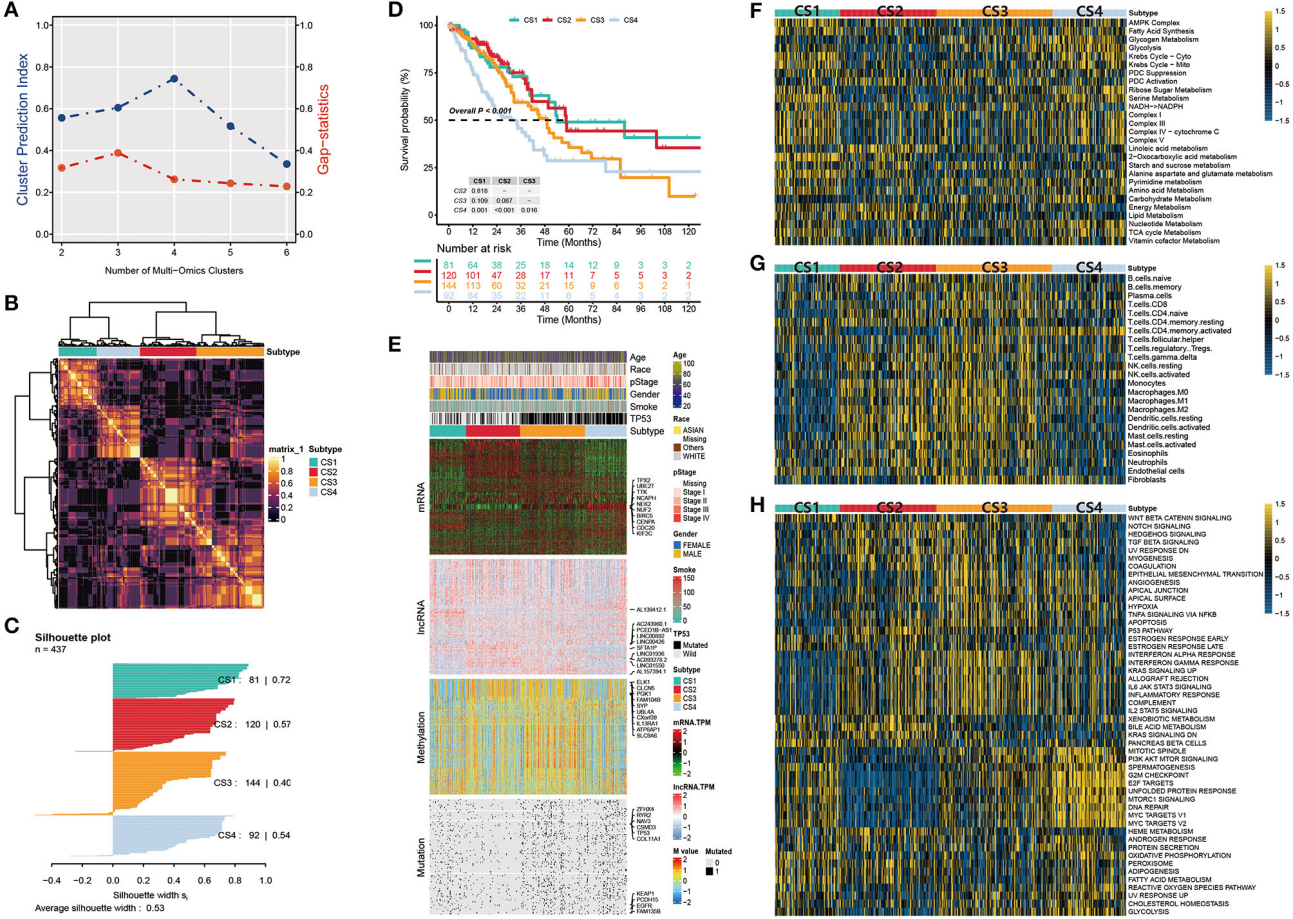
**(A)** Identification of the optimal number of clusters by calculating CPI (blue line) and the gap statistic (red line) in the LUAD cohort. **(B)** Consensus heatmap based on the results of five multi-omics comprehensive clustering algorithms with a cluster number of 4. **(C)** Silhouette scores were used to quantify sample similarity based on the consensus clustering results. **(D)** Kaplan-Meier survival analysis of overall survival in the four subtypes. **(E)** Comprehensive heatmap of multi-omics integrative clustering by five clustering algorithms with annotation of the top features. **(F)** Heatmap of specific metabolism-related pathways in the four subtypes. **(G)** Heatmap of specific immune-related pathways in the four subtypes. **(H)** Heatmap of specific tumor-associated pathways in the four subtypes.

After matching multiple omics data, 437 samples were included in the follow-up analysis. First, we estimated the number of clusters based on CPI and gaps statistics analysis. The CPI peaked at 4 and the gap statistic did not decline too much at a *k* value of =4; therefore, the optimal number of four clusters was considered (Figure 2A). Subsequently, to make the classification more robust, we integrated the clustering results of the five algorithms using a consensus ensemble (Figure 2B). Ultimately, the patients were divided into four subtypes: CS1, CS2, CS3, and CS4. We also quantified the similarity of the samples through silhouette analysis. The results showed that these subtypes were well separated and distinguishable from each other, with silhouette scores of 0.72, 0.57, 0.40, and 0.54, respectively (Figure 2C). The survival analysis showed that the four subtypes had significantly different OS rates (*P* < 0.001). The prognosis of CS1 and CS2 was relatively favorable, whereas that of CS3 and CS4 was relatively poor. Among the four subtypes, CS4 had the worst prognosis, and its OS was significantly lower than those of other subtypes (CS4 vs. CS1, *P* = 0.001; CS4 vs. CS2, *P* < 0.001; CS4 vs. CS3, *P* = 0.016; Figure 2D).

The distribution of the multi-omics data for each subtype is shown in Figure 2E. The heatmap of the mRNA showed that each subtype was clearly distinguished. A relatively obvious hypermethylated region was observed in the CS3 subtype. Additionally, the CS3 and CS4 subtypes were far more mutated than the other two subtypes. Within the top 10 representative omics datasets, we found that TPX2, UBE2T, TTK, NCAPH, and NEK2 were the top mRNA features, and TP53, EGFR, KEAP1, RYR2, and CSMD3 were the top mutations with a high impact on subtyping. PCED1B-AS1, SFTA1P, LINC00892, LINC00426 and ELK1, CLCN5, PGK1, SYP were the representative lncRNA and genes corresponding to the methylated sites, respectively. Moreover, information on patient age, race, pathological stage, sex, smoking history, and TP53 mutations were also listed.

### Clinical Characteristics

To characterize the basic information of LUAD patients, we summarized the clinical variables of the four patient subtypes, including age, race, pathological stage, sex, and TP53 (Table 1). In this cohort of 437 patients, we found a significant difference in patient age; the CS2 group seemed to be enriched in elderly patients (*P* = 0.004). The CS2 and CS3 subtypes had more female patients, while the CS1 and CS4 subtypes had more male patients (*P* < 0.001). In addition, the TP53 mutations were significantly higher in CS3 and CS4 than in CS1 and CS2 patients (*P* < 0.001).

**Table 1:** Baseline characteristics of LUAD participants in the CS1, CS2, CS3 and CS4.

	**CS1 (*N* = 81)**	**CS2 (*N* = 120)**	**CS3 (*N* = 144)**	**CS4 (*N* = 92)**	***P*** ^a^
**Age [median (IQR)]**	65.00 (60.00, 71.00)	69.00 (61.00, 75.00)	63.00 (56.00, 72.00)	63.00 (58.00, 72.00)	0.004
**Gender (%)**					<0.001
FEMALE	32 (39.5)	79 (65.8)	88 (61.1)	36 (39.1)	
MALE	49 (60.5)	41 (34.2)	56 (38.9)	56 (60.9)	
**Race (%)**					0.632
ASIAN	1 (1.4)	3 (2.7)	2 (1.5)	0 (0.0)	
**Others**	11 (15.7)	11 (9.8)	15 (11.5)	13 (15.1)	
WHITE	58 (82.9)	98 (87.5)	114 (87.0)	73 (84.9)	
**pStage (%)**					0.040
Stage I	44 (54.3)	78 (66.1)	74 (52.1)	41 (45.1)	
Stage II	17 (21.0)	23 (19.5)	44 (31.0)	22 (24.2)	
Stage III	15 (18.5)	13 (11.0)	20 (14.1)	21 (23.1)	
Stage IV	5 (6.2)	4 (3.4)	4 (2.8)	7 (7.7)	
**TP53 (%)**					<0.001
Mutated	21 (25.9)	33 (27.5)	107 (74.3)	73 (79.3)	
Wild	60 (74.1)	87 (72.5)	37 (25.7)	19 (20.7)	

a *χ^2^ or Fisher's exact test for categorical data and Mann-Whitney test for continuous data*.

### Signaling Pathway Analysis in the Four Subtypes

Tumors reprogram the metabolic pathway to meet the requirements for biosynthesis and nutrition, and metabolic patterns are associated with prognosis in many cancers (42, 43). Therefore, further understanding cancer metabolism and identifying key pathways may reveal the pathogenesis of lung adenocarcinoma and improve clinical treatment. Thus, we first studied whether there were different metabolic characteristics among the subtypes. In the TCGA-LUAD cohort, we estimated the GSVA enrichment score of the metabolic pathway and constructed a heatmap for visualization (Figure 2F). We found that each subtype had unique metabolic pathways with different metabolic levels. Among these, CS1 and CS4 were enriched in more metabolic pathways. The Krebs cycle ribose, sugar metabolism, serine metabolism, and other metabolic pathways were highly activated. In contrast, we found that carbohydrate and nucleotide metabolism pathways associated with poor prognosis were upregulated in CS3 and CS4, respectively. This is consistent with the hypothesis that cancer cells have increased requirements for glucose uptake and nucleotide synthesis (29, 44). In contrast, the lipid metabolic pathway is associated with a better prognosis and is upregulated in CS1 and CS2 (45).

We then explored the enrichment of immune-related pathways in LUAD subtypes to assess their immunological status and presented them in a heatmap (Figure 2G). Based on their immune infiltration, we observed that CS2 and CS3 were immune-hot subtypes. This implies that these patients may have a better response to immunotherapy. These subtypes had higher infiltration of monocytes, macrophages, dendritic cells, and some B and T cells. In addition, CS3 and CS4 were enriched in the fibroblast pathway, which may lead to a poor prognosis for patients with these two subtypes.

We further compared the activation status of 50 tumor-associated pathways among the four subtypes (Figure 2H). The results showed that the mitotic spindle, PI3K/AKT/mTOR signaling, G2M checkpoint, E2F targets, unfolded protein response, mtorc1 signaling, DNA repair, and two MYC target pathways were highly activated in CS4 cells. These activated cell cycle-and oncogenic-related pathways were positively correlated with the poor prognosis of CS4 and were significantly downregulated in CS2. Epithelial-mesenchymal transition (EMT) is a key process in cancer cell metastasis. In this process, epithelial cells acquire the characteristics of mesenchymal cells, which enhances the mobility and migration ability of cancer cells. The epithelial-mesenchymal transition pathway is activated in CS3 and CS4, which may be related to poor prognosis. We also observed that the interferon alpha response, interferon gamma response, allograft rejection, IL-2-STAT5 signaling, IL-6/JAK/STAT3 signaling, inflammatory response, and other pathways were activated in CS2 and CS3. These results suggest that these two subtypes are related to immune response and inflammation. Simultaneously, these immune-related pathways were significantly downregulated in CS1 mice. Although both the CS2 and CS3 subtypes showed activation of immune-related pathways, more oncogenic pathways in CS3 were activated, including EMT, angiogenesis, hypoxia, apoptosis, and the PI3K/AKT/mTOR signaling pathway, which may contribute to shaping a poor prognostic molecular subtype of CS3.

### The Effect of Genetic Alteration on Subtypes

**Figure 3:**
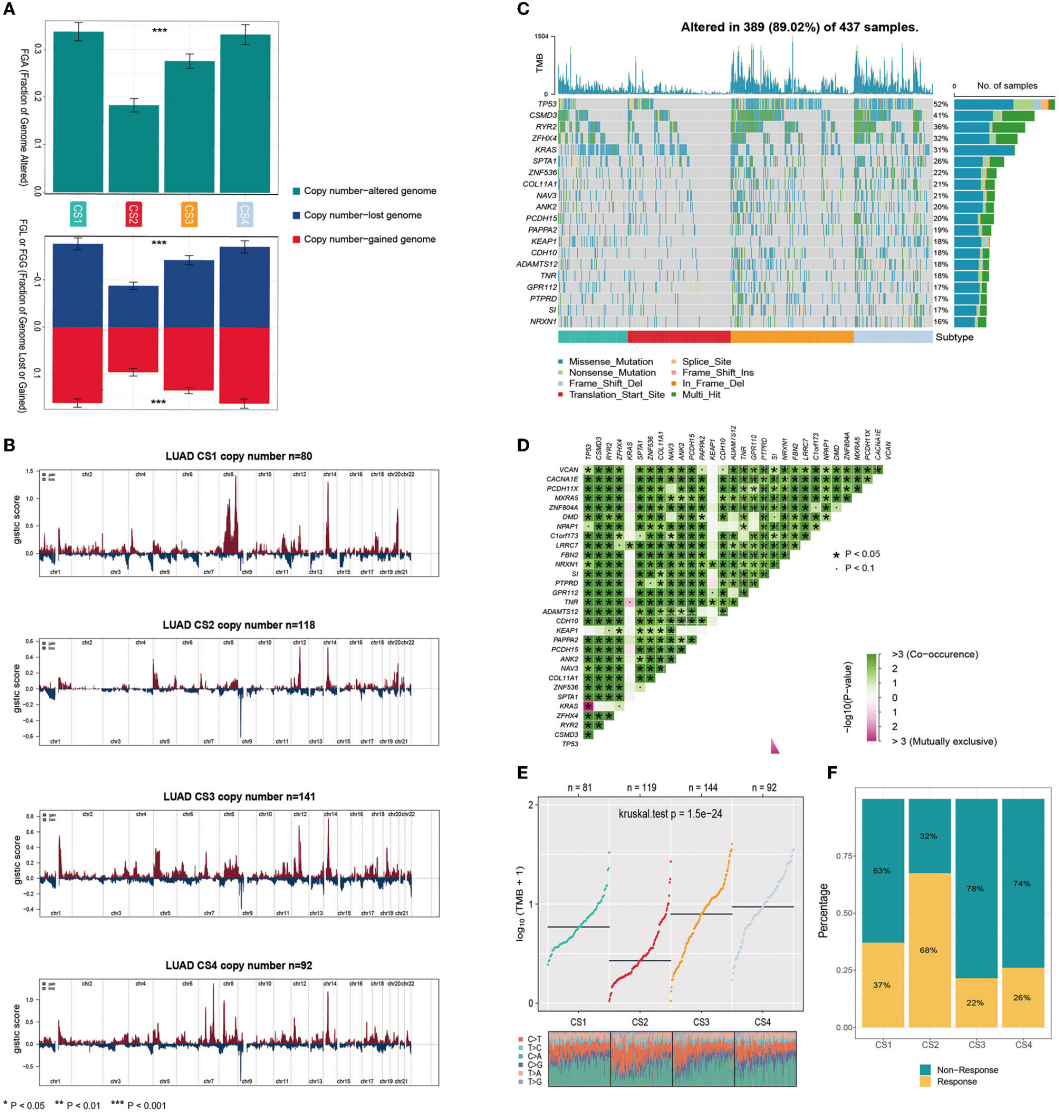
**(A)** Bar plot of fraction genome altered among the four subtypes. **(B)** The copy number amplifications and deletions among the 22 chromosomes in the four subgroups. **(C)** The waterfall plot shows the somatic mutation landscape of the top 15 most frequently mutated genes. The bar plot above the heatmap denotes the number of mutations occurring for each subject and the right side bar plot shows the number of subjects having a mutation for each gene. **(D)** The heatmap shows the mutually co-occurring and exclusive mutations of the top 30 frequently mutated genes. **(E)** Comparison of TMB and TiTv (transitions and transversions) among the four subtypes. **(F)** The bar plot of immunotherapy responders and non-responders predicted by the TIDE method.

Gene mutations and copy number alterations play key roles in tumorigenesis and cancer development. Therefore, we compared genetic alterations between the subtypes. We first evaluated the copy number alterations of the four subtypes and found that, compared with the other three subtypes, CS2 had fewer CNAs in both lost and gained genomes (Figure 3A). To explore the difference in SCNA among the patients of different subtypes, we used GISTIC 2.0 to analyze the changes in their chromosomal regions and drew copy number amplification and deletion maps according to the G score (Figure 3B). We found that CS1 had obvious copy number amplification on chromosomes 8, 14 and 20. CS4 had obvious copy number amplification on chromosomes 7, 8 and 14, and it showed copy number deletion on chromosomes 9. Although CS2 and CS3 showed similar copy number variation regions, the cell band of C3 had more regional amplification and deletion than C2, which may also be the reason why C2 was better than the C3 subtype in terms of prognosis. In short, alterations in copy number may be among the mechanisms that lead to differences in metabolism, immunity, and prognosis among the four subtypes.

Gene function is not only affected by its expression level and copy number variation but also by mutations. As shown in Figure 3C, the analysis of somatic mutations showed that 389 of 437 (89.02%) lung adenocarcinoma patients had mutations, most of which were missense ones. Compared to the CS1 and CS2 subtypes, CS3 and CS4 had a higher number of mutant genes. Twenty mutant genes had an overall mutation rate of >15%. TP53 had the highest mutation frequency, with mutations occurring in 52% of the samples. TP53, CSMD3, RYR2, ZFHX4, and KRAS were the top five mutated genes in lung adenocarcinoma samples. TP53 and KRAS are common gene mutations in lung adenocarcinoma and often indicate a worse prognosis. More KEAP1 mutations were also present in CS1 and CS4; studies have shown that non-small cell lung cancer (NSCLC) patients carrying STK11/KEAP1 mutations have less immune cell infiltration, which may lead to a poorer response to immune checkpoint inhibitors (ICIs) or poorer survival. We analyzed the mutual exclusion and co-occurrence of mutations. Most mutations co-occurred, whereas KRAS was mutually exclusive with TP53 (Figure 3D).

The tumor mutational burden (TMB) has become a promising and clinically validated biomarker for immune checkpoint inhibitors. We observed significantly higher average TMB values for CS3 and CS4 (*P* < 0.001, Figure 3E). Tumors with high TMB levels represent a potentially high number of neoantigens in tumor cells that can be recognized by the immune system. They are more likely to be recognized by the immune system and respond to immunotherapy. However, they may be affected by the immune microenvironment. The results of our evaluation using the TIDE algorithm showed that patients of the CS2 subtype were more likely to respond positively to immunotherapy (Figure 3F).

### Differential Analysis of Two Immune Subtypes

**Figure 4:**
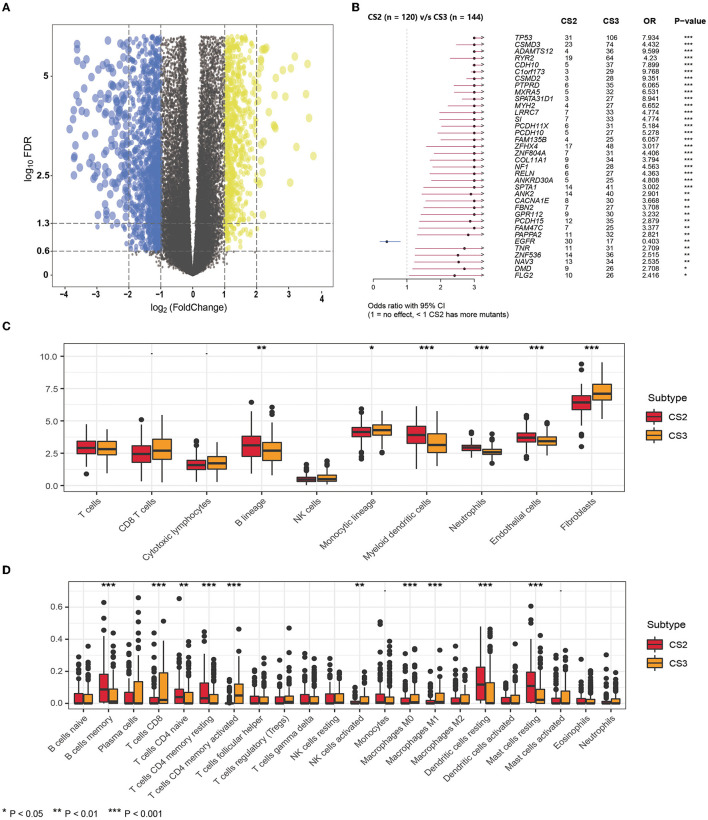
**(A)** The volcano plot shows the overall pattern of differentially expressed genes in the CS2 and CS3 subgroups. **(B)** The forest plot displays the significantly differentially mutated genes between two subgroups. **(C)** The boxplot shows the abundance of ten immune infiltrating cells calculated by the MCP-counter algorithm in different subtypes. **(D)** The boxplot shows the infiltration of LM22 immune cells evaluated by the CIBERSORT method in two subtypes.

CS2 and CS3 are two potential immune subtypes; however, their immune infiltration profiles and potential responses to immunotherapy are different and require further analysis. Therefore, we further compared the CS2 and CS3 subtypes. First, we performed differential expression analysis of CS2 and CS3 by DESeq2 and identified 585 significantly differentially expressed genes (Figure 4A), with a threshold value of FDR < 0.05, and an absolute value of log2FC > 2. This included 255 genes that were significantly upregulated and 330 genes that were significantly downregulated in the CS2 subtype (Supplementary Table S4). Among these genes, nine (i.e., TFF2, REG4, TFF1, ANXA10, MUC17, EPS8L3, MUC5AC, ONECUT3, and GC) were upregulated by more than five times, and three (i.e., MAGEA6, MAGEA4, and MAGEB2) were downregulated by more than five times. Among the significantly downregulated genes, the MAGE-A subfamily members play an important role in patient prognosis. Their overexpression is linked to poor prognosis in lung cancer and could serve as a potential prognostic marker (46). This finding is consistent with the differences in survival between CS2 and CS3.

In addition, we found significant differences in mutations between CS2 and CS3 (Figure 4B), where CS3 harbored more mutated genes, but EGFR mutations were more frequent in CS2 than in CS3. This implies that such patients may benefit from EGFR-targeted therapy.

Next, we compared differences in immune cell infiltration. We used the MCP-counter algorithm to calculate the abundance of 10 immune-infiltrating cells (Figure 4C), demonstrating that the immune enrichment score of CS2 in the B lineage, myeloid dendritic cells, neutrophils, and endothelial cells was significantly higher than that of CS3. However, CS3 cells had higher monocytic lineage and fibroblast cell enrichment score. In lung adenocarcinoma, infiltration by B cells could independently predict favorable prognosis (47), whereas fibroblasts were reported to be associated with poor outcomes (30, 48), which is consistent with the poorer prognosis in CS3.

In addition, we used the CIBERSORT method to evaluate the infiltration of LM22 immune cells (Figure 4D; Supplementary Table S5) and found that although the CS2 subtype had higher infiltration of B cell memory, in CD4 naive T cells, CD4 memory resting cells, dendritic cells, and resting mast cells, the CS3 subtype appeared to exhibit a more highly activated immune infiltration. Specifically, the CS3 subtype showed high expression in terms of T cells CD8, T cells CD4 memory activated, and activated NK cells. At the same time, CS3 also showed higher infiltration of the anti-inflammatory immune cell macrophages M0 and M1.

### Precision Treatment Recommendations for LUAD Patients

**Figure 5:**
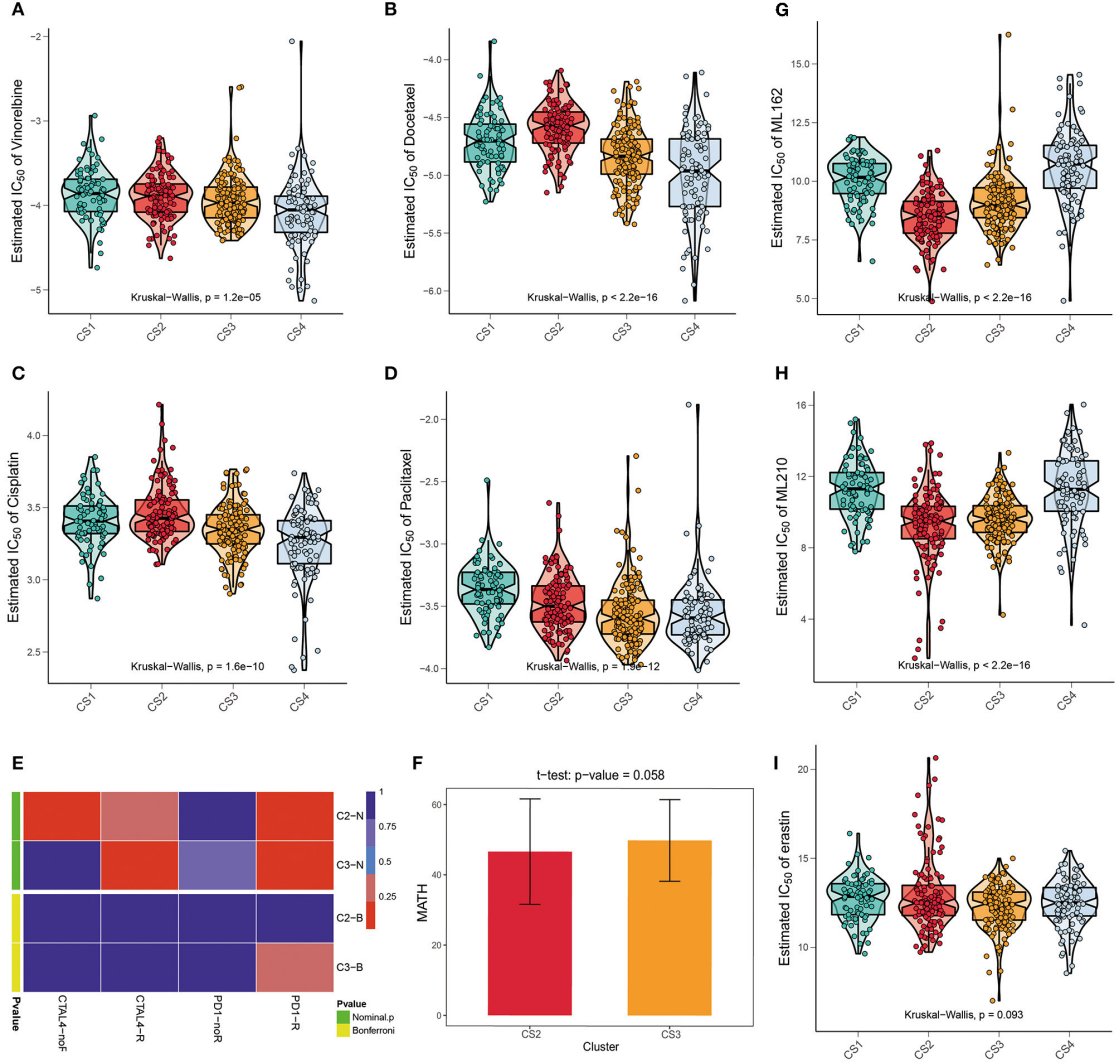
**(A–D)** The box plots of the estimated IC_50_ for common chemotherapy drugs (cisplatin, paclitaxel, docetaxel, and vinorelbine) of lung adenocarcinoma between the four subtypes. **(E)** Submap analysis manifested that patients in CS3 were more likely to respond to anti-PD1-R immunotherapy. **(F)** The t test of the MATH value revealed a difference in intra-tumoral heterogeneity between CS2 and CS3. **(G–I)** The box plots of the estimated IC_50_ for three ferroptosis inhibitors, ML162, ML210, and erastin among the four subtypes.

**Figure 6:**
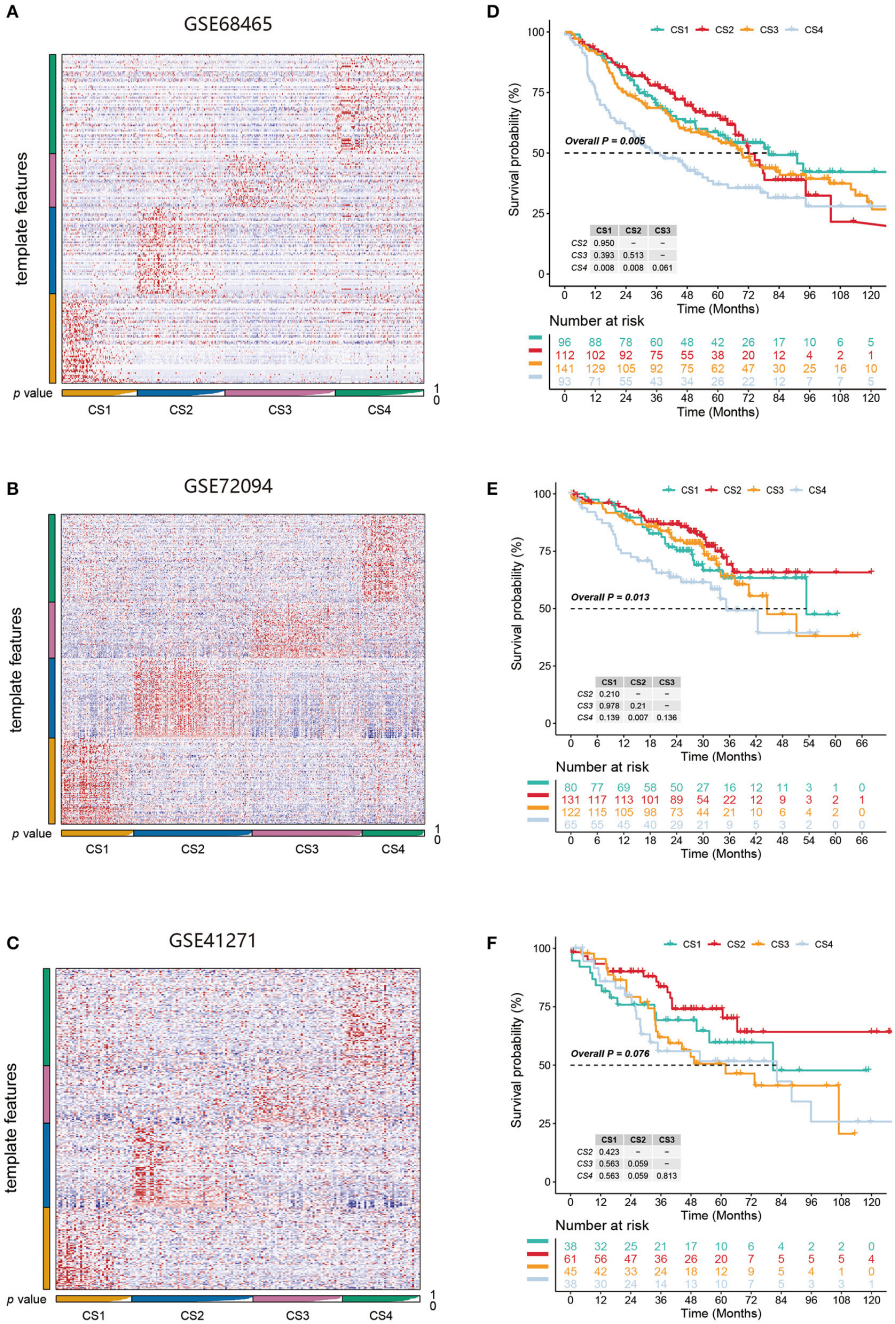
**(A–C)** Heatmap of NTP in three external cohorts GSE68465, GSE72094, and GSE41271 using subtype-specific upregulated biomarkers identified from the LUAD cohort. **(D–F)** Kaplan-Meier survival curve of the predicted four subtypes of three external cohorts.

We referred to the chemotherapy regimen for cell lung cancer in the NCCN Guidelines (Version 3.2020) and analyzed the drug sensitivity of common chemotherapy drugs for lung adenocarcinoma, including cisplatin, paclitaxel, docetaxel, and vinorelbine (Figures 5A–D). We trained the prediction model on the GDSC cell line dataset using ridge regression and assessed the accuracy of the prediction by ten-fold cross-validation, based on which the IC_50_ values of each sample in each subtype were estimated. As shown in Figure 6, CS3 and CS4 patients had lower IC_50_ values, indicating that they may be more sensitive to these chemotherapy drugs. Previous studies have shown that inhibition of STAT3 expression may be the key to the combination of paclitaxel and cisplatin in NSCLC chemotherapy (49–51). miR-526b-3p promotes the response to cisplatin by inhibiting STAT3. miR-9600 enhanced the drug sensitivity of non-small cell lung cancer to paclitaxel and cisplatin by inhibiting targeted STAT3. In our study, STAT3 expression was significantly lower in CS3 and CS4 [CS1: 159.11 (126.81, 199.74); CS2: 147.22 (129.03, 182.49); CS3: 129.68 (108.29, 167.60); CS4: 122.66 (98.27, 159.05); *P* < 0.001], which may be the main reason why these two subtypes are more sensitive to cisplatin and paclitaxel. On the other hand, class III β-tubulin (TUBB3) is a predictive marker of vinorelbine sensitivity in non-small cell lung cancer (52). Tumors with high levels of TUBB3 expression were more sensitive to vinorelbine. This is also consistent with our findings [CS1: 4.58 (2.10, 10.14); CS2: 2.27 (0.89, 3.85); CS3: 7.78 (3.75, 13.19); CS4: 8.47 (3.89, 14.13); *P* < 0.001]. Although previous studies have shown that inhibitor of growth 4 (ING4) is associated with docetaxel sensitivity (53), no such association was found in our subtypes. There may be other confounding factors, or other potential markers yet to be discovered.

We further explored the possibility of the response of the two groups of patients with potential immune subtypes to immunotherapy. First, we made predictions using the TIDE algorithm and found that 81 patients in CS2 and 31 patients in CS3 had a potential response to immunotherapy (Figure 3F; 68 and 22%, respectively; *P* < 0.001). We then compared the expression profiles of the two immune subtypes in LUAD with another published dataset of patients with melanoma responding to immunotherapy to predict the clinical response to immune checkpoint blockade using the subtype mapping method (Figure 5E). The results showed that CS3 patients were more likely to respond to anti-PD-1 immunotherapy (*P* = 0.015). TP53 missense mutations are associated with better clinical outcomes of anti-PD-1/L1 therapy, and patients with TP53/KRAS double mutations are a superior population for immunotherapy. Therefore, CS3 may be more likely to respond to anti-PD-1 immunotherapy than the other subtypes. In addition to TIDE and SubMap predictions, we also used intra-tumoral heterogeneity to predict the effect of immunotherapy. Studies have shown that intra-tumoral heterogeneity can better predict immunotherapy outcomes. The higher the intra-tumoral heterogeneity, the easier it is to suppress the antitumor immune response. We used the MATH value as a biomarker of intra-tumoral heterogeneity and quantified intra-tumoral heterogeneity using the ratio of the width of the mutant allele component in the tumor-specific mutation locus to the central distribution (Supplementary Table S6). Although the T test results showed that the intra-tumoral heterogeneity in CS2 and CS3 was not significantly different (*P* = 0.058), the ITH in CS2 was lower (Figure 5F), with a trend of responding better to immunotherapy.

Recently, ferroptosis has gained popularity as an alternative therapy for malignancies resistant to conventional therapies (54). A correlation was found between the KEAP1 mutation status and erastin-induced or ML162-induced ferroptosis. KEAP1 mutations are associated with resistance to ferroptosis (55). We performed drug sensitivity analysis of the three main ferroptosis inhibitors, ML162, ML210, and erastin. The results showed that ML162 and ML210 were less sensitive to CS1 and CS4 (Figures 5G,H; *P* < 0.001). Although the difference in drug sensitivity to erastin was not significant, there was a trend for it to be lower in CS1 and CS4 (Figure 5I; *P* = 0.093).

### Validation of Subtypes in Three External Cohorts

We identified the first 200 upregulated biomarkers in the CS1, CS2, CS3, and CS4 subtypes using “DESeq2,” adjusted for a significance threshold of *P* < 0.05. Three external cohorts GSE68465, GSE72094, and GSE41271 were used to verify the reliability of the new subtype. Predictions were made in each cohort using the NTP method based on the specific upregulation of biomarkers in the subtypes (Figures 6A–C). It is worth noting that in the three external validation cohorts, although the survival differences of the CS1, CS2, and CS3 subtypes did not approach statistical significance, we observed some prognostic predictions that were consistent with those of the original subtypes (Figures 6D–F). Specifically, the survival analysis showed that the clinical prognosis of CS4 was the worst among all subtypes, while the prognosis of patients with the CS2 subtype was the most favorable (GSE68465, *P* < 0.005; GSE72094, *P* = 0.013; GSE41271, *P* = 0.076).

## Discussion

Lung cancer is a fatal malignancy and a leading cause of cancer-related mortality worldwide. With the development of high-throughput biochemical technologies, a large amount of omics data has been accumulated to help characterize the molecular mechanisms of different types of cancers (56). The high heterogeneity and complex molecular patterns of LUAD make it difficult to analyze marker genes or mutations alone, and a more comprehensive understanding of the molecular mechanisms of LUAD is necessary. Multi-omics data provide a valuable resource for subtype analysis. Previous studies have identified LUAD subtypes based on single omics datasets or a single clustering algorithm (7, 16). However, few studies have combined multi-omics data with multiple clustering algorithms to classify LUAD (57). However, such analyses are often limited. It is difficult to obtain a comprehensive understanding of tumor occurrence and progression for analysis using a single omics dataset. Additionally, a single clustering algorithm may not be robust. Therefore, integrating multi-omics information provides more correlational evidence for biological mechanisms, thus allowing for a deeper understanding of complex biological processes. In our study, we combined five clustering algorithms to derive stable and robust subtypes.

To better understand the molecular characteristics of LUAD, we classified it into four subtypes. We speculated that CS1 and CS4 may be less responsive to immunotherapy. In addition, CS4 had more mutations and a worse prognostic profile than CS1. The immune infiltration profiles of CS2 and CS3 differed. In contrast, CS3 harbored more mutations with remarkably poor clinical outcomes. The functional and signaling pathway enrichment analyses confirmed that CS1 was enriched in more metabolic pathways than CS4, but was barely enriched in immune-related pathways, while fewer oncogenic pathways were activated. Both the CS2 and CS3 subtypes showed activation of immune-related pathways, but more oncogenic pathways were activated in CS3. In addition to metabolism-related pathways, the CS4 subtype was also enriched in cell cycle-related, oncogenic, and malignant fibroblast pathways.

Our study not only classifies LUAD patients but also provides new insights for predicting the sensitivity of immunotherapy and chemotherapy and possible targeted therapies. Recently, molecular targeted therapy has significantly improved the treatment of cancer patients. Epidermal growth factor receptor (EGFR) is the most representative mutation in LUAD patients and can be used as a sensitive therapeutic target inhibitor of tyrosine kinase (TKI) (58). In our study, patients belong to CS2 had more EGFR mutations (0 vs. 25.00% vs. 11.81% vs. 7.61%; *P* < 0.001); thus, CS2 could be a potential population that were benefit from targeted therapy. Additionally, KRAS had a higher mutation frequency in our finding, especially in the CS1 subtype, with significantly more KRAS mutations (55.56% vs. 26.67% vs. 27.78% vs. 20.65%; *P* < 0.001; Supplementary Table S7). This may lead to unfavorable response of chemotherapy in these patients, and may affect the efficacy of EGFR-TKI. However, the CS2 subtype may show higher likelihood of responding to the KRAS inhibitor (e.g., Sotorasib) as compared to other subtypes (59).

Immune checkpoint inhibitors (ICIs) have emerged as one of the most promising approaches for cancer therapy (60). Studies have shown that immune checkpoint inhibitor therapy targeting PD-L1/PD-1 is a promising solution in the field of NSCLC treatments (61). Some treatments have been approved by the FDA for the treatment of advanced NSCLC patients and have shown significant efficacy in clinical practice (62, 63). We used the SubMap method to predict the likelihood that CS3 patients would be more responsive to anti-PD-1 immunotherapy. In CS3 patients, who are rich in TP53 mutations and may respond to PD-1 immunotherapy, this treatment may have better efficacy (64). Although immune checkpoint inhibitors seem promising for lung cancer treatment, not all lung cancer patients respond to immune checkpoint inhibitor targeting, probably because of the complexity and limitations of tumor immunity. Therefore, LUAD subtyping may reveal subgroups of patients that could benefit from immunotherapy or chemotherapy. We used TIDE predictions and found that CS2 was a more promising subtype for responding to immunotherapy. These results suggest that the CS2 subtype has a better immune infiltration environment and may therefore inhibit tumor growth, progression, invasion, and metastasis, thus demonstrating a better prognosis. Although CS3 did not have a high response rate to immunotherapy in TIDE prediction, high TP53 mutations and highly activated immune infiltration made it likely to benefit from anti-PD-1 therapy.

In addition, considering that chemotherapy remains a common method for the treatment of lung cancer, we estimated the chemosensitivity of each sample based on the IC_50_ value. The results showed that subtypes CS3 and CS4 were more sensitive to chemotherapy than the other two subtypes.

Ferroptosis affects the efficacy of chemotherapy, radiotherapy, and immunotherapy; therefore, combinations of drugs that target ferroptosis signaling could improve the outcomes of these therapies. We investigated the sensitivity of different subtypes of major ferroptosis inhibitors. We found that CS1 and CS4, which are enriched for KEAP1 mutations, are less sensitive to ferroptosis inhibitors and may be less likely to benefit from them.

Our study is the first to evaluate LUAD classifications based on multi-omics data and multi-clustering methods. A comprehensive molecular characterization analysis of LUAD was performed. Molecular differences between the identified subtypes may be beneficial in providing new markers for specific treatments and opening new avenues for precision therapy in lung adenocarcinoma. Our immune prediction and drug sensitivity analysis also provide potential therapeutic strategies for immunotherapy and chemotherapy. Meanwhile, the prominent molecular features of each subtype may guide the development of new drug strategies. However, this study has some limitations. First, our study was retrospective; therefore, our results need to be confirmed by prospective experiments. In addition, TCGA data included in the analysis were mostly from patients in developed countries, and data from developing countries were lacking.

## Data Availability Statement

Raw data for this study were generated at TCGA with cancer type of LUAD. And the validation datasets can be found in the GEO data repository. The datasets and codes used during the current study are available from the online repositories https://github.com/ruan2ruan/Multi-omics-Analysis-of-LUAD.

## Author Contributions

FY and XL designed and guided this work. JZ and YC supervised this work. XR participated in data collecting, data processing, program implementation, and paper writing. LX and WC contributed to statistical analysis. YY and MH contributed to paper writing and article polish. All authors provided critical advice for the final manuscript.

## Funding

This work was supported by the Key R&D Program of Jiangsu Province (Social Development) (BE2020694) and the National Natural Science Foundation of China (81973145).

## Conflict of Interest

The authors declare that the research was conducted in the absence of any commercial or financial relationships that could be construed as a potential conflict of interest.

## Publisher's Note

All claims expressed in this article are solely those of the authors and do not necessarily represent those of their affiliated organizations, or those of the publisher, the editors and the reviewers. Any product that may be evaluated in this article, or claim that may be made by its manufacturer, is not guaranteed or endorsed by the publisher.
